# Epidemiology of rhegmatogenous retinal detachment in commercially insured myopes in the United States

**DOI:** 10.1038/s41598-023-35520-x

**Published:** 2023-06-09

**Authors:** Cassie A. Ludwig, Daniel Vail, Ahmad Al-Moujahed, Natalia F. Callaway, Namrata Saroj, Andrew Moshfeghi, Darius M. Moshfeghi

**Affiliations:** 1 Department of Ophthalmology, Byers Eye Institute, Stanford University, Palo Alto, CA 94304 USA; 2 Department of Ophthalmology, New York Eye and Ear Infirmary of Mount Sinai, New York, NY USA; 3 Retina Department, Massachusetts Eye and Ear, Harvard Medical School, Boston, MA USA; 4 All Eyes Consulting, LLC, New York, NY USA; 5 Department of Ophthalmology, USC Eye Institute, Keck School of Medicine of USC, University of Southern California, 1975 Zonal Ave, Los Angeles, CA 90033 USA

**Keywords:** Eye diseases, Retinal diseases

## Abstract

Myopia is a known risk factor for rhegmatogenous retinal detachment (RRD). Given global trends of increasing myopia, we aimed to determine the absolute risk (incidence rate) of RRD in non-myopes, myopes and high myopes in the United States over ten years. We performed a retrospective cohort study of 85,476,781 commercially insured patients enrolled in the Merative™ Marketscan^®^ Research Database. The incidence rate of RRD in phakic patients in the United States was 39-fold higher in high myopes than non-myopes (868.83 per 100,000 person-years versus 22.44 per 100,000 person-years) and three-fold higher in myopes than non-myopes (67.51 per 100,000 person-years versus 22.44 per 100,000 person-years). The incidence rate was significantly higher in males in each category (*P* < 0.01). Combined, the incidence rate of RRD in phakic patients in the United States from 2007 to 2016 was 25.27 RRDs per 100,000 person-years, a rate higher than those in prior published studies in North America, South America, Europe, Asia, and Australia. The absolute risk of myopia and high myopia increased from 2007 to 2016. The risk of RRD in phakic high myopes rose with increasing age. Notably, the magnitude of increased risk of RRD in myopes varied substantially according to the minimum follow-up period in our models and should be accounted for when interpreting data analyses.

## Introduction

Rhegmatogenous retinal detachments (RRDs) are separations of the neurosensory retina from the retinal pigment epithelium that occur following a break in the retina. In 80–90% of RRDs, the precipitating event is a retinal break associated with a posterior vitreous detachment (PVD)^1^. The relationship between myopia, PVDs and RRDs has long been known^2^. Myopia accelerates PVD formation independent of aging. In myopia, increased posterior segment volume exceeds production of vitreous gel components resulting in amplified traction of the vitreous gel on both the macula and the vitreous base. Myopic traction maculopathy, consisting of foveal retinoschisis, macular hole, and/or foveoschisis retinal detachment (FRD) occurs in 9.0% to 34.3% of highly myopic eyes with posterior staphyloma^3,4^.

In addition to its effect on the posterior retina, an increase in axial length can alter the structure of the anterior retina. An increase in axial length leads to a proportional increase in risk of RRD. Researchers consistently find a higher risk of RRD following cataract extraction in high myopes with Lin et al. reporting a 43-fold higher risk in high myopes compared to non-myopes^5,6^. This is thought to be secondary to an increase in peripheral vitreoretinal degenerative pathology associated with anomalous vitreoretinal adhesions in myopes. Lattice degeneration, for example, yields exaggerated vitreoretinal adhesions at the margin of the lesion and is more common with an increase in axial length^7^. Such taut adhesions increase the risk of tear with traction. Lattice degeneration (51%) and white without pressure (12%) were highly prevalent in an adult population of high myopes in Hong Kong ^8^.

A recent study by Willis et al. reported that nearly 4 percent of US adults have high myopia by applying prevalence calculations from 2005 to 2008 NHANES data to estimates from the US Population Census in 2014 ^9^.Given this elevated prevalence of high myopia and the risk of vision loss associated with RRD, we aimed to determine the incidence rate of RRD in phakic non-myopes, myopes and high myopes in a large population of commercially insured US patients. Herein, we additionally estimate the incidence rate and period prevalence of RRDs, myopia, and high myopia in phakic commercially insured patients in the US. We hypothesized that the incidence rate of RRD is rising along with the rising prevalence of high myopia. Knowledge of the absolute risk and incidence of RRDs is necessary for optometrists and ophthalmologists to prepare for the rising burden of myopia.

## Materials and methods

### Study population

Our study population consists of 123,637,719 commercially insured patients in the Merative™ Marketscan^®^ Research Database^10^ from 2007 to 2016 with outpatient encounters. Stanford University grants access to the Merative™ Marketscan^®^ Research Database through the Population Health Services portal. The Merative™ Marketscan^®^ Research Database contains de-identified records of patients with employer-sponsored health insurance and contains insured medical claims from beneficiaries from all 50 US states. The Merative™ Marketscan^®^ Research Database includes diagnostic and procedural information of individual-level health care claims from both inpatient and outpatient encounters.

We selected all patients in the Merative™ Marketscan^®^ Research Database who were continuously enrolled in the database, and who had at least one year of enrollment. We excluded patients with non-continuous enrollment to avoid including patients in our analysis who may have received care during the study period that was not accounted for in the database. We excluded patients from our analysis if they carried any diagnosis of sickle cell, serous retinal detachment, tractional retinal detachment, chorioretinitis, ruptured globe, choroidal hemorrhage, retinoschisis, vitreous hemorrhage or pseudophakia. Patients were classified as having myopia or high myopia if they carried a relevant *International Classification of Diseases* diagnosis code (ICD 9 367.1, ICD 10 H52.1 for myopia and ICD 9 360.21, ICD 10 H44.2* for high myopia) at any point during their enrollment. Patients with RRD were identified by the date of first diagnosis code for any RRD (ICD 9 36101-36105, ICD 10 H330*).

### Ethics

This secondary analysis of de-identified data is not classified as human subjects research and is exempt from institutional review board approval at Stanford University. This analysis conformed to the principles of the Declaration of Helsinki and Health Insurance Portability and Accountability Act regulations.

### Statistical analysis

The primary outcome was the absolute risk of RRD in myopes and high myopes, as indicated by ICD codes. To estimate an unadjusted incidence of RRD, we constructed life tables by calculating the number of new diagnoses of RRD in each year of our sample and dividing total RRD diagnoses by the number of days of enrollment in that year preceding an RRD diagnosis (if any) for each patient. Once patients discontinued enrollment in the database or were diagnosed with the outcome (RRD), they were excluded from the denominator in calculations of incidence in subsequent years. The confidence interval (CI) of the incidence rate was calculated based on the Poisson distribution and the Z test was used to examine the difference in the incidence of RRD incidence between the comparison groups. To calculate estimates of patient risk for RRD that were adjusted for age, sex, and myopia status, we specified time-to-event survival models measuring time from patients’ enrollment in the database until their first diagnosis of RRD, or, if they were never diagnosed with RRD, until they discontinued enrollment in the database. Specifically, we specified Cox proportional hazards models to calculate the risk of RRD given age, gender, myopia, and high myopia diagnosis, allowing for interaction terms between each of those variables. As a sensitivity analysis, we re-estimated these models under increasingly stringent requirements for observed time in the database. We estimated models that required all patients in the sample to have at least one year of observed data, models that required patients to have two years of data, and so on up to a maximum of ten years of observed data. These increasingly restrictive requirements for observed follow-up time do limit the sample of patients that can be included in each subsequent model, but also give us more confidence that the RRD diagnoses we observe in patients records really are “new” diagnoses of RRD, as opposed to diagnostic codes that pre-dated patients’ enrollment in the database. All data were analyzed using Stata 14.2 (StataCorp, College Station, TX, US).

## Results

### Sample descriptives

Of 85,476,781 commercially insured patients who met inclusion and exclusion criteria, 52.9% were female (n = 45,243,736) (Table 1). Average age at the beginning of coverage was 32.6 years (SD 18.7 years) and average age at diagnosis of RRD was 51.9 years (SD 12.1 years). From 2007 to 2016, a total of 106,823 (0.12%) patients carried a diagnosis of RRD, 1,896,659 (2.22%) carried a diagnosis of myopia, and 108,158 (0.13%) of high myopia (i.e., period prevalence).

**Table 1:** Characteristics of patients enrolled in the study.

Characteristics	All patients (n = 85,476,781)
Gender [n, (%)]	
Male	40,233,045 (47.07)
Female	45,243,736 (52.93)
Age at beginning of coverage (yrs) [mean (SD)]	32.56 (18.70)
Age at RRD diagnosis (yrs) [mean (SD)]	51.92 (12.13)
Diagnosis [n, (%)]	
RRD	106,823 (0.12)
Myopia	1,896,659 (2.22)
High myopia	108,158 (0.13)

*RRD* rhegmatogenous retinal detachment, *SD* standard deviation.

### Incidence rate of rhegmatogenous retinal detachment, myopia, high myopia

**Figure 1:**
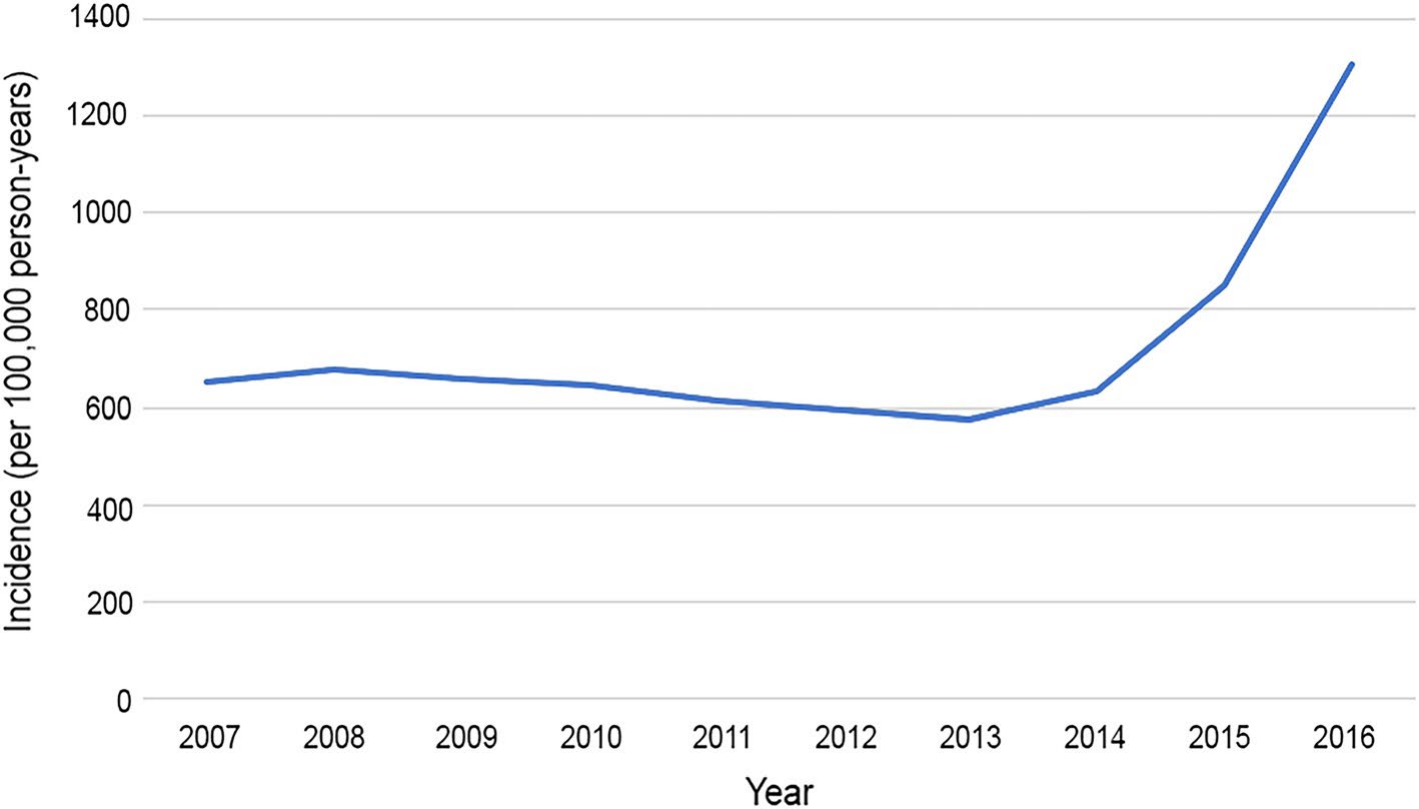
Myopia incidence rate per 100,000 person-years. The incidence of myopia in the cohort declined slightly from 2007 to 2013 then increased sharply from 2013 to 2016.

**Figure 2:**
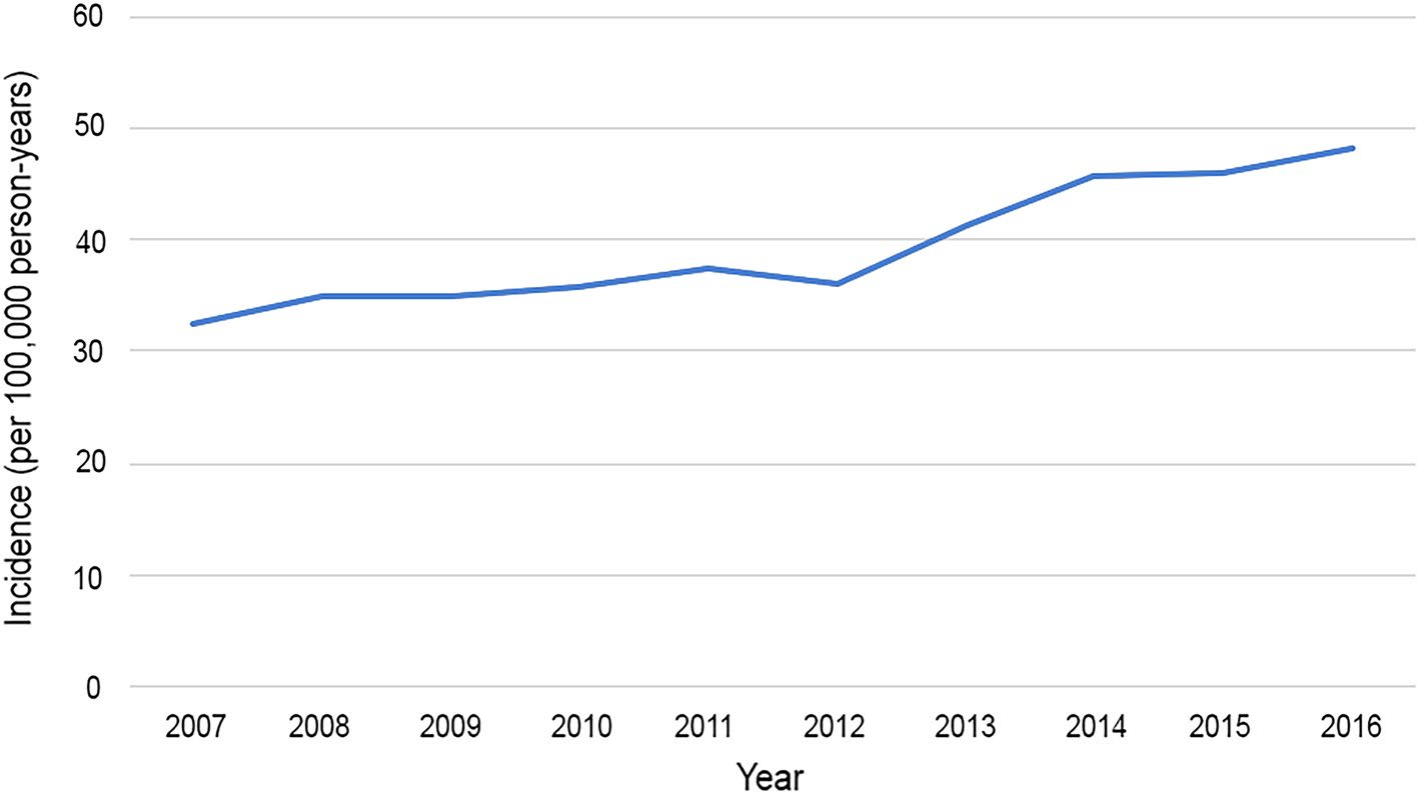
High myopia incidence rate per 100,000 person-years. The incidence of high myopia in the cohort gradually increased from 2007 to 2016.

The incidence rate of RRD ranged from 21.72 (95% CI 21.09, 22.35) to 33.60 (95% CI 32.84, 34.36) RRDs per 100,000 person-years from 2007 to 2016 (Table 2). The rate of myopia minimally declined from 653.93 (95% CI 650.56, 657.30) myopes per 100,000 person-years in 2007 to 630.77 (95% CI 627.95, 633.59) myopes per 100,000 person-years in 2014 (*P* < 0.01), followed by a rapid increase to 1,307.19 (95% CI 1301.99, 1312.39) myopes per 100,000 person-years in 2016 (*P* < 0.01) (Fig. 1, Supplementary Table 1). The rate of high myopia steadily increased from 32.42 (95% CI 31.67, 33.17) high myopes per 100,000 person-years in 2007 to 48.09 (95% CI 47.10, 49.08) high myopes per 100,000 person-years in 2016 (*P* < 0.01) (Fig. 2, Supplementary Table 2).

**Table 2:** Incidence of rhegmatogenous retinal detachment (RRD) per 100,000 person-years in the Merative™ Marketscan® Research Database between 2007 and 2016.

Year	Number of patients	Mean days at risk	Person-years at risk	New diagnosis of RRD	Incidence of RRD per 100,000 person-years	95% confidence interval
2007	22,163,189	340.43	20,671,272.41	6,945	33.60	32.84–34.36
2008	33,841,550	332.26	30,806,408.00	8,471	27.50	26.94–28.06
2009	36,170,889	331.85	32,886,146.16	8,114	24.67	24.16–25.18
2010	35,906,604	329.82	32,446,230.47	7,743	23.86	23.35–24.37
2011	37,921,042	327.43	34,017,273.32	8,472	24.90	24.40–25.40
2012	38,189,162	329.20	34,443,286.50	8,276	24.03	23.54–24.52
2013	32,489,301	327.95	29,191,761.56	6,889	23.60	23.07–24.13
2014	30,990,326	332.40	28,222,788.00	6,137	21.74	21.22–22.26
2015	21,083,952	329.56	19,036,867.78	4,135	21.72	21.09–22.35
2016	18,959,621	345.23	17,932,736.76	5,675	31.65	30.85–32.45

### Incidence rate of rhegmatogenous retinal detachment in non-myopes, myopes, high myopes

**Figure 3:**
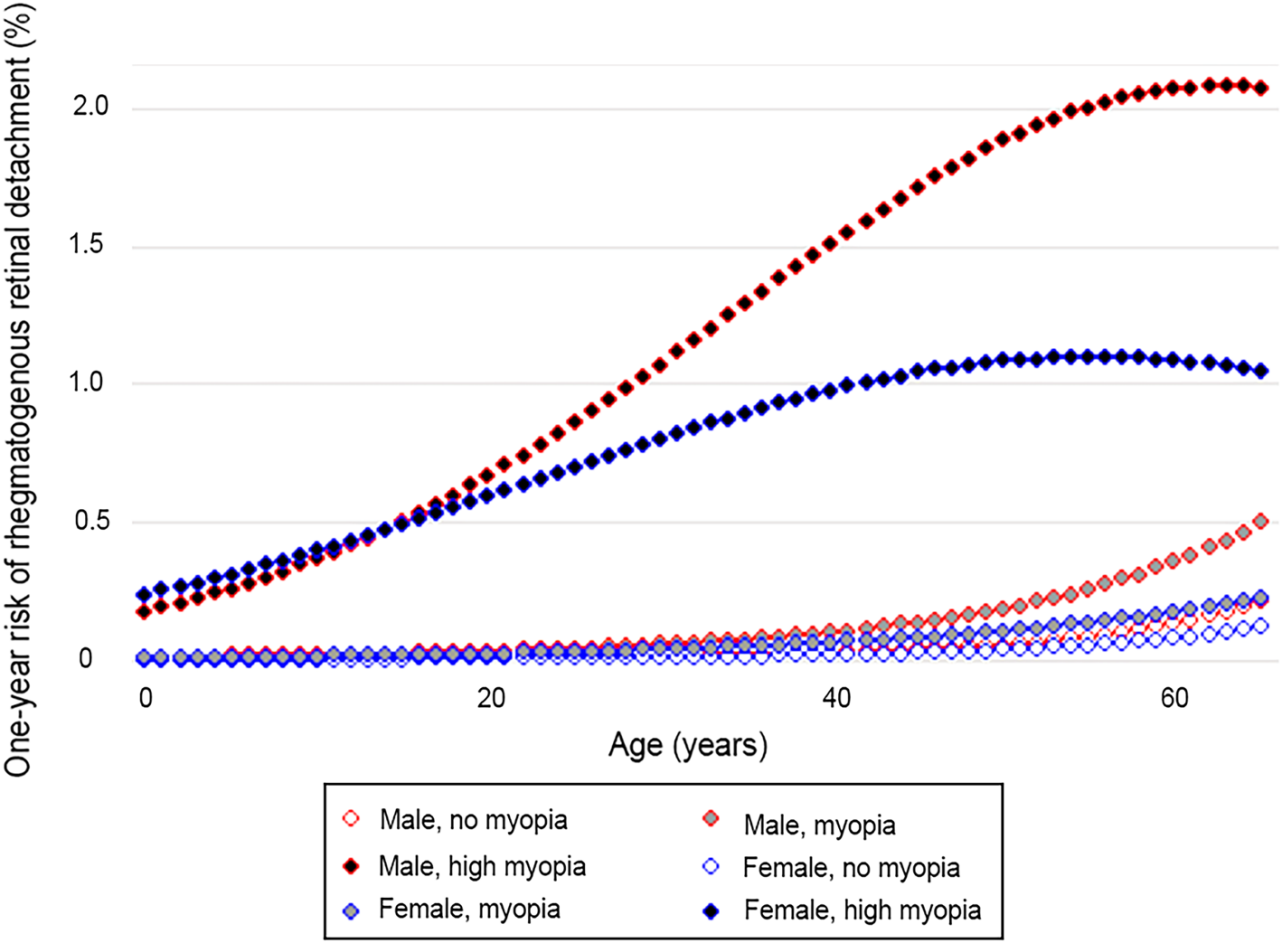
Annual risk of rhegmatogenous detachment for men and women of different ages, stratified by myopia status. Men appear to be at higher risk of RRD after adjusting for age and myopia status.

The overall incidence rate of RRD pooling across all years from 2007 to 2016 was 25.27 (95% CI 24.93, 25.60) RRDs per 100,000 person-years (30.19, 95% CI 29.65, 20.73 in males, 20.81, 95% CI 20.39, 21.23 in females, *P* < 0.01). Non-myopes had the lowest incidence rate of 22.44 (95% CI 22.12, 22.76) RRDs per 100,000 person-years, myopes had an incidence rate of 67.51 (95% CI 63.81, 71.21) RRDs per 100,000 person-years, and high myopes had the highest incidence rate of 868.83 (95% CI 813.28, 924.38) RRDs per 100,000 person-years. Male non-myopes (27.08, 95% CI 26.57, 27.59 per 100,000 person-years), myopes (89.85, 95% CI 83.08, 96.62 per 100,000 person-years), and high myopes (1116.62, 95% CI 1014.20, 1219.04 per 100,000 person-years) had a higher incidence rate than females in each category (non-myopes: 18.20 per 100,000 person-years, 95% CI 17.80, 18.60, *P* < 0.01; myopes: 52.42 per 100,000 person-years, 95% CI 48.19, 56.65, *P* < 0.01; high myopes: 718.41 per 100,000 person-years, 95% CI 654.36, 782.46, *P* < 0.01) (Fig. 3).

### Risk of rhegmatogenous retinal detachment under models with different follow-up periods

We recalculated this model under different minimum follow-up periods, increasing up to ten years. The estimated risk of RRD for men and women, with and without high myopia, is plotted for each of these ten models in the Supplementary Fig. 1, to illustrate how sensitive the estimates are to minimum follow-up period. With one-year follow-up, the absolute risk of RRD in high myopes was 1433.92 and 918.91 per 100,000 person-years in males and females, respectively. This decreased to 568.47 and 446.62 per 100,000 person-years with a minimum ten-year follow-up.

## Discussion

Myopia contributes significantly to the risk of RRD. We found a three-fold higher incidence rate of RRDs in myopes as compared to non-myopes and a 39-fold higher incidence rate of RRDs in high myopes as compared to non-myopes. Male non-myopes, myopes, and high myopes all had a higher annual risk of RRD as compared to females in each category.

The known risk factors for RRD include older age^11^, myopia/longer axial length^11–16^, a history of trauma^17^, vitreoretinal degenerations^12^, male sex^18–20^, and occupational lifting^21,22^. The present study reinforces the importance of myopia and particularly high myopia as a risk factor for RRD along with age and male sex across non-myopes and myopes alike. PVDs are known to occur at a younger age and a higher frequency in myopes, leading to the leftward shift in the peak age of RRD in high myopes as compared to non-myopes herein (Fig. 3)^7,23^.

While several studies report on the prevalence of myopia within the population of those presenting with RRD, fewer describe the incidence rate of RRDs based on refractive error in a large population. Here we report an incidence rate of 22.44 RRDs per 100,000 person-years in non-myopes, 67.51 RRDs per 100,000 person-years in myopes, and 868.83 RRDs per 100,000 person-years in high-myopes. The risk in high myopes in this study exceeds even the postoperative risk reported by Williams et al. in 2008 who reported an incidence rate of 226.45 per 100,000 person-years in high myopes after cataract extraction^24^. The incidence rate also exceeds that described by T. C. Burton in 1989 who evaluated 172 residents of Iowa who developed RRDs that year and found that an emmetropic person without lattice had an annual risk of RRD of 2 in 100,000 people but that this increased 189-fold to 378 in 100,000 people in the presence of lattice and refractive error > − 5.0D^25^. While the relative risk (RR) for high myopes compared to non-myopes in the present study is lower (868.83/22.44 or 39-fold vs. 189/2 or 95-fold), the incidence rate in the population is more than fourfold higher than previously reported. This may be due to an increase in the extent of axial elongation since 1989. Alternatively, it is possible that isolated myopia and high myopia are underreported resulting in an ostensibly higher incidence rate. Healthy myopes may pay cash for their refractions whereas patients presenting for RRDs may receive the diagnosis of myopia or high myopia in addition to that for RRD.

Numerous studies have retrospectively investigated the frequency of RRD in patients undergoing refractive procedures including LASIK, phakic intraocular lenses, and phacoemulsification^26–30^. Sheu et al. found that high myopes, and more significantly male high myopes, may be at an increased risk for late post-operative RRD after cataract surgery^31^. In the study, cumulative risk of RRD increased with time, and increased dramatically after four years in male moderate and high myopes. In a recent review of RRD after phacoemulsification, Qureshi and Steel report six studies with a dose-dependent relationship between axial length and risk of pseudophakic RRD^32^. Lin et al. noted the greatest effect with axial length greater than 26 with a RR of 18.9, while axial length between 23 and 26 had a RR of 3.92 (as compared to those with an axial length of < 23 mm)^6^.

The influence of sex on the pathogenesis of myopia is complicated. Several studies report a higher mean axial length in men as compared to women (0.2 to 0.5 diopters depending on age category)^33–35^, and a higher rate of RRD in men as compared to women^18–20^. In our study, we report a higher prevalence of RRD in male non-myopes, myopes and high myopes as compared to females in each category. This is consistent with prior reports and is likely due to higher mean axial length. Dating back to 1903, Hertel et al. found that of 6,863 myopes presenting to his eye clinic, 71.5% were male, 85.9% of whom had presented with an RRD as compared to 73.1% of women^36^. In contrast to this and most other reports, Willis et al. found that upon multivariate analysis (adjusting for age and sex) the odds of high myopia was actually 1.7 times higher among women than men (adjusted OR, 1.66; 95% CI 1.24–2.24)^9^. However, as the authors report, this was based on NHANES autorefraction data which may overestimate myopia in younger populations and older populations with myopic shift secondary to cataracts^9^. Another study in Taiwan by Chen et al. found a higher incidence of RRDs among women aged 20–29 as compared to males, though again overall more men than women were diagnosed with RRDs in the setting of myopia (male: female ratio 0.79)^18^. Similarly, we found a higher incidence of RRDs in one small subset—young females. Though, the overall incidence of RRDs in those under age 10 is low, as with previously reported ranges of 0.4 to 2.9 cases per 100,000 in this population^19,20,36–40^.

Prior studies report a difference in age distribution of RRDs between Western countries with a single peak in the fifth to sixth decade of life^11,41^, and Eastern countries with a peak in the second decade of life (RRD related to high myopia) and sixth decade of life (RRD related to PVD)^18,40,42–44^. In our study, as in those before, we report a consistent overall average peak of RRDs in the fifth decade of life among high myopes with a slightly later peak occurring in non-myopes, though the data is limited for patients > 65 years of age who are primarily covered by Medicare rather than commercial insurance in the US.

The incidence rate of RRDs reported varies by age, race, and geographical origin of those studied. We report a higher overall incidence rate of RRDs in this population of commercially insured phakic patients in the United States (pooled: 25.7 per 100,000 person-years, 2016: 31.7) as compared to prior published studies in North America (9.1 to 12.4 per 100,000 person-years)^37,45^, South America (9.2 per 100,000 person-years)^19^, Europe (6.3 to 17.9 per 100,000 person-years)^11,27,41,46–48^, Asia (8.0 to 16.4 per 100,000 person-years)^18,20,40,42,49,50^, and the Australian continent (11.8 per 100,000 person-years)^51^. The period prevalence (2007 to 2016) of RRD in the present study was 0.12%, while that of myopia was 2.22% and of high myopia was 0.13%. This is lower than prevalence reported in recent studies, but also captures a specific subset of the population—commercially insured phakic patients under 65 years of age. ^52^ More importantly, as in other studies, RRD^53^, myopia^54^, and high myopia^9^ appear to be on the rise.

Though the Merative™ Marketscan^®^ Research Database is not weighted to be nationally representative, it includes approximately 50% of those receiving health insurance through an employer and therefore represents a large subset of insured Americans under the age of 65. Therefore, this study is well-suited to detect rising trends in the US population. While many studies have examined the incidence of RRD in the US, few have specifically looked at incidence rate of RRD in high myopes and myopes. This study maintains standard limitations associated with use of a retrospective commercial claims data, namely, reliance on accurate diagnostic coding. Providers may code for myopia rather than high myopia even when the latter diagnosis is more accurate. Providers may also have arbitrary cut-offs for myopia and high myopia, increasing the possibility of mixed cohorts. Additionally, coding for high myopia does not always indicate axial myopia as refractive status is dependent on the optical power of the cornea, aqueous humor, lens, and vitreous humor^54^. The slow decline in myopia incidence followed by a rapid rise within this study window is more likely to reflect changes in reimbursement for coding for myopia than an actual change in incidence across the population. However, assuming accurate diagnoses, this highlights prior underdiagnosis of myopia even if only for reimbursement reasons. Furthermore, pseudophakia was an exclusion criterion in this study as myopia and high myopia are commonly defined by providers using spherical equivalent and spherical equivalent is modified following cataract surgery. This may bias the results toward an underestimate of the absolute risk of RRD in myopia given the propensity for myopes to develop cataracts at younger ages. Specific to the Merative™ Marketscan^®^ Research Database—race data is not included. This is important as prior studies have highlighted discrepancies in risk and characteristics of RRDs across various races.^55,56^.

Understanding the epidemiology of RRDs in myopia and high myopia in the United States is critical for eye care providers to better monitor high risk populations. RRDs can be debilitating. We report on average a 39-fold higher incidence rate of RRDs in high myopes as compared to non-myopes, and an average higher incidence rate of RRDs in the fourth to sixth decade of life. Further studies are needed to explore the pathophysiological root behind sex differences in predisposition to RRD.

## Supplementary Information

Supplementary Figure S1.

Supplementary Table S1.

Supplementary Table S2.

## Data Availability

The data that support the findings of this study are available from Merative™ Marketscan^®^ Research Database but restrictions apply to the availability of these data, which were used under license for the current study, and so are not publicly available. Data are however available from the authors upon reasonable request and with permission of Merative™ Marketscan^®^ Research Database.

## References

[1] Michels, R., Wilkinson, C. & RIce, T. *Michels Retinal Detachment*. (Mosby Inc., 1997).

[2] Donders, F. C. *Ametropie en Hare Gevolgen*. (C. van der Post, Jr, 1860).

[3] Takano M, Kishi S (1999). Foveal retinoschisis and retinal detachment in severely myopic eyes with posterior staphyloma. Am. J. Ophthalmol..

[4] Baba T (2003). Prevalence and characteristics of foveal retinal detachment without macular hole in high myopia. Am. J. Ophthalmol..

[5] Neuhann IM (2008). Retinal detachment after phacoemulsification in high myopia: Analysis of 2356 cases. J. Cataract Refract. Surg..

[6] Lin J-Y, Ho W-L, Ger L-P, Sheu S-J (2013). Analysis of factors correlated with the development of pseudophakic retinal detachment - A long-term study in a single medical center. Graefes Arch. Clin. Exp. Ophthalmol..

[7] Pierro L, Camesasca FI, Mischi M, Brancato R (1992). Peripheral retinal changes and axial myopia. Retina.

[8] Lam D (2005). Prevalence and characteristics of peripheral retinal degeneration in chinese adults with high myopia: A cross-sectional prevalence survey. Optom. Vis. Sci..

[9] Willis JR (2016). The prevalence of myopic choroidal neovascularization in the United States. Ophthalmology.

[10] Stanford Center for Population Health Sciences. (2019). MarketScan Databases (Version 2.0). Redivis. 10.57761/ray7-1g16.

[11] Mitry D (2010). The epidemiology and socioeconomic associations of retinal detachment in Scotland: A two-year prospective population-based study. Investig. Opthalmol. Vis. Sci..

[12] Chen S, Jiunn-Feng H, Te-Cheng Y (2006). Pediatric rhegmatogenous retinal detachment in Taiwan. Retina.

[13] Chou S-C (2006). Characteristics of primary rhegmatogenous retinal detachment in Taiwan. Eye.

[14] Wang N-K (2005). Pediatric rhegmatogenous retinal detachment in East Asians. Ophthalmology.

[15] Chang P-Y (2005). Clinical characteristics and surgical outcomes of pediatric rhegmatogenous retinal detachment in Taiwan. Am. J. Ophthalmol..

[16] Spitznas M, Graeff V (1970). Netzhautablösung und refraktion. DMW - Deutsche Medizinische Wochenschrift.

[17] Cox MS, Schepens CL, Freeman HM (1966). Retinal detachment due to ocular contusion. Arch. Ophthalmol..

[18] Chen S-N, Lian I-B, Wei Y-J (2015). Epidemiology and clinical characteristics of rhegmatogenous retinal detachment in Taiwan. Br. J. Ophthalmol..

[19] Limeira-Soares PH, Lira RPC, Arieta CEL, Kara-José N (2006). Demand incidence of retinal detachment in Brazil. Eye.

[20] Incidence and epidemiological characteristics of rhegmatogenous retinal detachment in Beijing, China. *Ophthalmology* **110**, 2413–2417 (2003). 10.1016/s0161-6420(03)00867-4 14644727

[21] Sheu S-J, Ger L-P, Chen J-F (2007). Male sex as a risk factor for pseudophakic retinal detachment after cataract extraction in Taiwanese adults. Ophthalmology.

[22] Farioli A, Kriebel D, Mattioli S, Kjellberg K, Hemmingsson T (2017). Occupational lifting and rhegmatogenous retinal detachment: A follow-up study of Swedish conscripts. Occup. Environ. Med..

[23] Seabag, J. Myopia effects upon vitreous-significance in retinal detachments. In *Anterior and Posterior Segment Surgery: Mutual Problems and Common Interests* 366–372 (Ophthalmic Communications Society, 1998).

[24] Williams MA (2009). The incidence and rate of rhegmatogenous retinal detachment seven years after cataract surgery in patients with high myopia. Ulster Med J.

[25] Burton TC (1989). The influence of refractive error and lattice degeneration on the incidence of retinal detachment. Trans. Am. Ophthalmol. Soc..

[26] Bjerrum SS, Mikkelsen KL, La Cour M (2013). Risk of pseudophakic retinal detachment in 202 226 patients using the fellow nonoperated eye as reference. Ophthalmology.

[27] Bechrakis NE, Dimmer A (2018). Rhegmatogene Netzhautablösung. Ophthalmologe.

[28] Chan C (2004). Characteristics of sixty myopic eyes with pre-laser in situ keratomileusis retinal examination and post-laser in situ Keratomileusis retinal lesions. Retina.

[29] O’Brien TP, Awwad ST (2002). Phakic intraocular lenses and refractory lensectomy for myopia. Curr. Opin. Ophthalmol..

[30] Srinivasan B (2016). Modern phacoemulsification and intraocular lens implantation (refractive lens exchange) is safe and effective in treating high myopia. Asia-Pac. J. Ophthalmol..

[31] Sheu S-J, Ger L-P, Ho W-L (2010). Late increased risk of retinal detachment after cataract extraction. Am. J. Ophthalmol..

[32] Qureshi MH, Steel DHW (2020). Retinal detachment following cataract phacoemulsification—a review of the literature. Eye (Lond).

[33] Lim LS (2010). Distribution and determinants of ocular biometric parameters in an Asian population: The Singapore Malay eye study. Investig. Opthalmol. Vis. Sci..

[34] Fotedar R (2010). Distribution of axial length and ocular biometry measured using partial coherence laser interferometry (IOL Master) in an older white population. Ophthalmology.

[35] Asakuma T (2012). Prevalence and risk factors for myopic retinopathy in a Japanese population. Ophthalmology.

[36] Hertel E (1903). Über Myopie. Albrecht von Græfe’s Archiv für Ophthalmologie.

[37] Haimann MH, Burton TC, Brown CK (1982). Epidemiology of retinal detachment. Arch. Ophthalmol..

[38] Fivgas G, Capone A (2001). Pediatric rhegmatogenous retinal detachment. Retina.

[39] Rosner M, Treister G, Belkin M (1987). Epidemiology of retinal detachment in childhood and adolescence. J. Pediatr. Ophthalmol. Strabismus.

[40] Wong TY (1999). Racial difference in the incidence of retinal detachment in Singapore. Arch. Ophthalmol..

[41] Van de Put MAJ, Hooymans JMM, Los LI (2013). The incidence of rhegmatogenous retinal detachment in The Netherlands. Ophthalmology.

[42] Park SJ, Choi N-K, Park KH, Woo SJ (2013). Five year nationwide incidence of rhegmatogenous retinal detachment requiring surgery in Korea. PLoS ONE.

[43] Kim MS, Park SJ, Park KH, Woo SJ (2019). Different mechanistic association of myopia with rhegmatogenous retinal detachment between young and elderly patients. Biomed Res Int.

[44] Rim TH (2016). Refractive errors in Koreans: The Korea national health and nutrition examination survey 2008–2012. Korean J Ophthalmol.

[45] Wilkes SR, Beard CM, Kurland LT, Robertson DM, O’Fallon WM (1982). The incidence of retinal detachment in Rochester, Minnesota, 1970–1978. Am. J. Ophthalmol..

[46] Algvere PV, Jahnberg P, Textorius O (1999). The swedish retinal detachment register. Graefe’s Arch. Clin. Exp. Ophthalmol..

[47] Hajari JN (2014). A nationwide study on the incidence of rhegmatogenous retinal detachment in Denmark, with emphasis on the risk of the fellow eye. Retina.

[48] Mowatt L, Shun-Shin G, Price N (2003). Ethnic differences in the demand incidence of retinal detachments in two districts in the West Midlands. Eye.

[49] Zou H (2002). Epidemiology survey of rhegmatogenous retinal detachment in Beixinjing District, Shanghai. China. Retina.

[50] Sasaki K (1995). Epidemiologic characteristics of rhegmatogenous retinal detachment in Kumamoto, Japan. Graefes Arch. Clin. Exp. Ophthalmol..

[51] Polkinghorne PJ, Craig JP (2004). Northern New Zealand rhegmatogenous retinal detachment study: Epidemiology and risk factors. Clin. Experiment. Ophthalmol..

[52] Katz J, Tielsch JM, Sommer A (1997). Prevalence and risk factors for refractive errors in an adult inner city population. Invest. Ophthalmol Vis Sci.

[53] Nielsen BR, Alberti M, Bjerrum SS, Cour M (2020). The incidence of rhegmatogenous retinal detachment is increasing. Acta Ophthalmol..

[54] Holden BA (2016). Global prevalence of myopia and high myopia and temporal trends from 2000 through 2050. Ophthalmology.

[55] Chandra, A. *et al.* Ethnic variation in rhegmatogenous retinal detachments. *Eye (Lond).* **29**, 803–807 (2015). 10.1038/eye.2015.43 PMC4469671 25853394

[56] Rosman, M. *et al.* Retinal detachment in Chinese, Malay and Indian residents in Singapore: a comparative study on risk factors, clinical presentation and surgical outcomes. *Int Ophthalmol.* **24**, 101–106 (2001). 10.1023/a:1016306609978 12201344

