# Supplementary material for: Epidemiology of rhegmatogenous retinal detachment in commercially insured myopes in the United States
Source: Sci Rep. 2023 Jun 9;13:9430. doi: 10.1038/s41598-023-35520-x (PMC10256775; doi:10.1038/s41598-023-35520-x)
Supplement: Supplementary file 1 — Supplementary Figure S1. [file 41598_2023_35520_MOESM1_ESM.pdf]

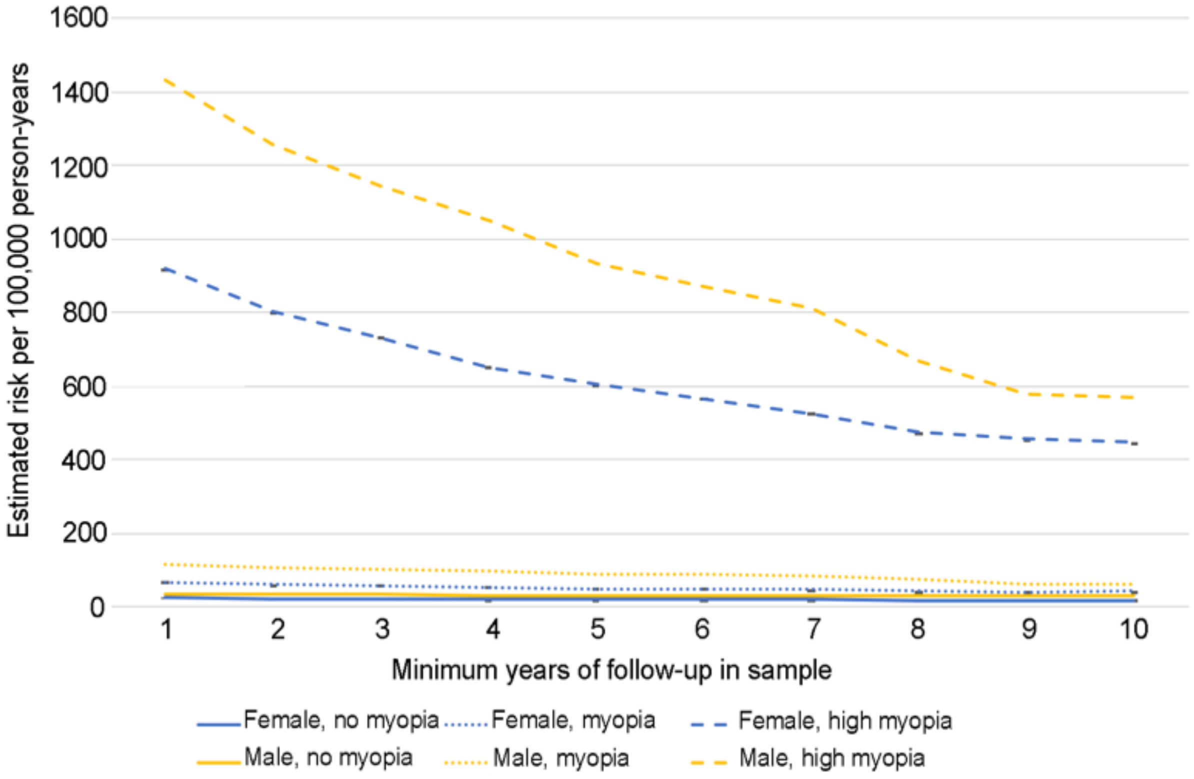

Supplementary Figure 1. Annual risk of rhegmatogenous retinal detachment for men and women with and without myopia, under models with different minimum follow-up periods (from 1-10 years).
