# Supplementary material for: Epidemiology of rhegmatogenous retinal detachment in commercially insured myopes in the United States
Source: Sci Rep. 2023 Jun 9;13:9430. doi: 10.1038/s41598-023-35520-x (PMC10256775; doi:10.1038/s41598-023-35520-x)
Supplement: Supplementary file 2 — Supplementary Table S1. [file 41598_2023_35520_MOESM2_ESM.docx]

| **Supplementary Table 1**. Incidence of Myopia per 100,000 Person-years in the IBM MarketScan Database between 2007 and 2016 | | | | | | |
| --- | --- | --- | --- | --- | --- | --- |
| Year | Number of Patients | Mean Days at Risk | Person-years at Risk | New Diagnosis of Myopia | Incidence of Myopia per 100,000 person-years | 95% Confidence Interval |
| 2007 | 22,163,406 | 339.45 | 20,611,767.20 | 134,787 | 653.93 | 650.56-657.30 |
| 2008 | 33,723,326 | 331.27 | 30,607,059.70 | 207,926 | 679.34 | 676.56-682.12 |
| 2009 | 35,916,977 | 330.79 | 32,550,296.70 | 213,982 | 657.39 | 654.74-660.04 |
| 2010 | 35,559,365 | 328.76 | 32,028,310.31 | 206,066 | 643.39 | 640.75-646.03 |
| 2011 | 37,501,054 | 326.48 | 33,543,788.67 | 205,096 | 611.43 | 608.93-613.93 |
| 2012 | 37,695,125 | 328.18 | 33,892,389.15 | 201,332 | 594.03 | 591.57-596.49 |
| 2013 | 32,047,247 | 326.90 | 28,702,401.20 | 165,755 | 577.50 | 574.87-580.13 |
| 2014 | 30,546,781 | 331.32 | 27,728,225.33 | 174,901 | 630.77 | 627.95-633.59 |
| 2015 | 20,764,265 | 328.35 | 18,679,384.88 | 158,667 | 849.42 | 845.46-853.38 |
| 2016 | 18,570,720 | 343.04 | 17,453,240.92 | 228,147 | 1307.19 | 1301.99-1312.39 |
