# Supplementary material for: Epidemiology of rhegmatogenous retinal detachment in commercially insured myopes in the United States
Source: Sci Rep. 2023 Jun 9;13:9430. doi: 10.1038/s41598-023-35520-x (PMC10256775; doi:10.1038/s41598-023-35520-x)
Supplement: Supplementary file 3 — Supplementary Table S2. [file 41598_2023_35520_MOESM3_ESM.docx]

| **Supplementary Table 2**. Incidence of High Myopia per 100,000 Person-years in the IBM MarketScan Database between 2007 and 2016 | | | | | | |
| --- | --- | --- | --- | --- | --- | --- |
| Year | Number of Patients | Mean Days at Risk | Person-years at Risk | New Diagnosis of High Myopia | Incidence of High Myopia per 100,000 person-years | 95% Confidence Interval |
| 2007 | 22,163,191 | 340.44 | 20,672,009.00 | 6,701 | 32.42 | 31.67-33.17 |
| 2008 | 33,841,767 | 332.25 | 30,805,613.47 | 10,790 | 35.03 | 34.40-35.66 |
| 2009 | 36,168,946 | 331.84 | 32,882,536.47 | 11,533 | 35.07 | 34.46-35.68 |
| 2010 | 35,902,335 | 329.80 | 32,440,346.61 | 11,573 | 35.67 | 35.05-36.29 |
| 2011 | 37,914,992 | 327.40 | 34,009,550.46 | 12,758 | 37.51 | 36.89-38.13 |
| 2012 | 38,179,944 | 329.17 | 34,432,472.66 | 12,435 | 36.11 | 35.51-36.71 |
| 2013 | 32,480,660 | 327.92 | 29,181,052.09 | 12,074 | 41.38 | 40.68-42.08 |
| 2014 | 30,978,340 | 332.36 | 28,208,214.40 | 12,930 | 45.84 | 45.09-46.59 |
| 2015 | 21,073,298 | 329.51 | 19,024,205.56 | 8,746 | 45.97 | 45.05-46.89 |
| 2016 | 18,946,890 | 345.20 | 17,919,288.55 | 8,618 | 48.09 | 47.10-49.08 |
